# Challenges in implementing integrated undergraduate medical curricula across low- and middle-income countries: A scoping review

**DOI:** 10.12669/pjms.42.9.16442

**Published:** 2026-09

**Authors:** Nadeem Razaq, Mohamad Nurman Yaman, Mohd Nasri Awang Besar, Suhaila Sanip

**Affiliations:** 1 Nadeem Razaq, MBBS, MMEd Associate Professor, Rai Medical College Sargodha, Pakistan Department of Medical Education Faculty of Medicine Universiti, Kebangsaan Malaysia (UKM) Kuala Lampur, Malaysia; 2 Mohamad Nurman Yaman, MBChB, MMEd, PhD Associate Professor, Department of Medical Education Faculty of Medicine Universiti, Kebangsaan Malaysia (UKM) Kuala Lumpur, Malaysia; 3 Mohd Nasri Awang Besar, MD, MMEd, PhD Associate Professor, Department of Medical Education, Sir Jeffery Cheah Sunway Medical School, Faculty of Medical and Life Sciences (FMLS), Sunway No. 5, Jalan Universiti, Bandar Sunway 47500 Selangor, Darul Ehsan, Malaysia; 4 Suhaila Sanip, MD, MMedEd, PhD Associate Professor, Medical Education Unit Faculty of Medicine and Health Sciences Universiti, Sains Islam Malaysia (USIM) Malaysia

**Keywords:** Curriculum, Developing Countries, Educational Measurement, Education, Faculty Development, Medical, Undergraduate

## Abstract

**Objectives:**

Integrated curricula have become a core element of undergraduate medical education (UGME) reforms worldwide, including low- and middle-income countries (LMICs). LMICs, which are often characterized as resource-constrained settings, encounter challenges in integrated curricula implementation. However, these challenges are reported fragmentarily. This review mapped the literature on the implementation of integrated curricula in LMICs, with attention to reported challenges, contextual variation and gaps in the evidence base.

**Methodology:**

Electronic searches were conducted between August 2025 and January 2026 at the Department of Medical Education, Faculty of Medicine, Universiti Kebangsaan Malaysia following the frameworks of Arksey and O’Malley and Levac et al. The literature published between January 2010 and December 2025 within LMIC medical schools was searched through PubMed, Scopus, Web of Science, EBSCO MEDLINE Complete and ERIC databases. Original peer-reviewed studies in English reporting the functional enactment of integrated undergraduate medical curricula were eligible for inclusion.

**Results:**

From 925 records identified, thirteen studies met the inclusion criteria. Six broad themes (with sub-themes) were identified: resources and infrastructure constraints (faculty shortages, lack of skills labs), governance and organizational barriers (policy-practice gaps, limited departmental autonomy), faculty development needs (role-transition resistance), assessment misalignment (exit exams undermining delivery), curriculum integration issues (content overload) and training settings constraints (rural supervision shortages). The findings indicated that while structural reforms are widespread, a deficiency in reporting on implementation outcomes remains a critical evidence gap.

**Conclusion:**

The findings suggest that the implementation of integrated UGME curricula in LMICs depends not only on curriculum design but also on the strength of institutional system and contextual readiness to support reforms into practice.

## INTRODUCTION

Integrated curricula represent a deliberate move away worldwide from conventional discipline-based models and have become a salient feature of undergraduate medical education (UGME) reforms.^1^ An integrated curriculum functions as a competency and system-based design where integration is an explicit structural feature. It demonstrates horizontal integration of relevant basic-science disciplines within the same phase of training as well as vertical integration connecting basic and clinical sciences across all years to promote continuity, contextual understanding, and early development of clinical reasoning.^2^ This framework has been progressively adopted in many lowand middleincome countries (LMICs) contemplating a broader transition towards competency-based education and advancing accreditation requirements.^3^ Notably, LMICs are classified by the World Bank based on Gross National Income (GNI) per capita.^4^

The implementation of an integrated curriculum, despite its pedagogical advantages, is inherently a complex process. It requires coordinated changes across faculty roles, assessment practices, organizational routines, and institutional support systems.^5^ Interestingly, the studies from LMICs advocate for the adoption of integrated curricula but have also reported specific challenges encountered during these transitions. However, these studies report these challenges in isolated components rather than examining the process comprehensively.^6^-^8^ Internationally, the curriculum reforms are increasingly viewed as occurring within complex adaptive systems, where changes in one component of the educational system, such as faculty roles, can have consequential effects on assessment practices, organizational structures, and learner engagement.^9^ This systemic interdependence is also mirrored at the national level; for instance, research in Pakistan demonstrates that the transition to modular integration is often hindered when institutional policies and faculty readiness are not aligned, reflecting the broader challenges faced across many LMICs.^10^ Curriculum implementation, thus, is not a set of separate problems to fix one by one. It operates as an interconnected system, where a change in one area inevitably affects others.^11^

Current evidence is often methodologically fragmented, with studies focus on student satisfaction or academic performance (outcomes) rather than on the organizational and systemic processes (governance, resource allocation, and faculty readiness) required to sustain the reforms.^12^ In addition, existing implementation frameworks are largely derived from high-income countries (HICs), creating a contextual gap. A consolidated synthesis, is therefore needed to clarify the nature of reported barriers, explore context led variations, and highlight areas where additional inquiry is obligatory. The aim of this scoping review was to map the empirical literature on the implementation of integrated UGME curricula in LMICs. The review, specifically charts reported challenges, examine context-specific variations, and identify existing literature gaps to produce a synthesized evidence-base that serves as a practical roadmap for educators and policymakers in resource-constrained settings.

## METHODOLOGY

This review was conducted at the Department of Medical Education, Faculty of Medicine, Universiti Kebangsaan Malaysia (UKM), from June 2025 to February 2026.

### Ethical Approval:

Formal ethical approval for the research was granted by the UKM Research Ethics Secretariat (Sekretariat Etika Penyelidikan UKM) under reference number JEP-2025-025 (dated: February 20, 2025).

It followed the methodological frameworks given by Arksey et al. and O’Malley et al.^13^ and Levac et al.^14^ that comprise of five key stages:

- identifying the research question,
- identifying relevant studies,
- selecting studies,
- charting the data, and
- collating and summarizing the results.

### Stage one: Identifying the research question:

Guided by the overarching aim and thereafter objectives of the study, three research questions were developed as follows:

**RQ1:** What types of implementation challenges are reported during the implementation of integrated UGME curricula in LMICs?

**RQ2:** What similarities and contextspecific variations in implementation challenges are reported across different LMIC settings?

**RQ3:** What gaps exist in the current literature regarding the implementation of integrated UGME curricula in LMICs?

### Stage two: Identifying relevant studies:

The following electronic databases and search engines were used between August 2025 and January 2026: PubMed, SciVerse Scopus, Web of Science Core Collection, EBSCO MEDLINE Complete, and the Education Resources Information Center (ERIC). Peer-reviewed, empirical studies published in indexed journals were searched. This approach is consistent with recent large-scale evidence syntheses in medical education, such as the scoping review by Alias et al. (2026)^12^, which prioritized peer-reviewed empirical data than grey literature when mapping 30 years of implementation trends. The search strategy combined controlled vocabulary terms (e.g., MeSH in PubMed) with free-text keywords, applied across titles, abstracts, and indexing fields. The core search terms were: “undergraduate medical education”, “integrated curriculum”, “medical curriculum reform”, “competencybased medical education”, “curriculum implementation”, “integration challenges”, “low- and middle-income countries”, and “LMICs”. (Appendix-A). The reference lists, in addition to all included studies were searched manually to relate any further publications.

### Stage three: Study selection:

The research team comprised the lead researcher (NR), principal supervisor (MNY), and two co-supervisors (MNAB, SS), all with advanced qualifications in medical education. Study selection was conducted collaboratively in two phases. Initially, a total of 925 records were identified across databases, which then reduced to 855 after the removal of duplicates. Two reviewers (NR and MNAB) independently screened the titles and abstracts, resulting in the exclusion of 833 irrelevant articles. Discrepancies between the two reviewers were forwarded to the principal supervisor (MNY), as senior third reviewer, for consensus and adjudication. The inter-rater reliability for initial screening phase using Cohen’s Kappa coefficient (k), yielded a strong agreement value of 0.84. NR and the fourth reviewer (SS) then evaluated the full texts of the twenty-two shortlisted articles collaboratively. During this, nine articles were excluded because they were not curriculum-focused, or focused solely on accreditation, teaching methods, or quality assurance systems. All four authors reached mutual agreement on the final inclusion of thirteen studies (Fig. 1). Studies were eligible if they met the following criteria:

**Fig.1:**
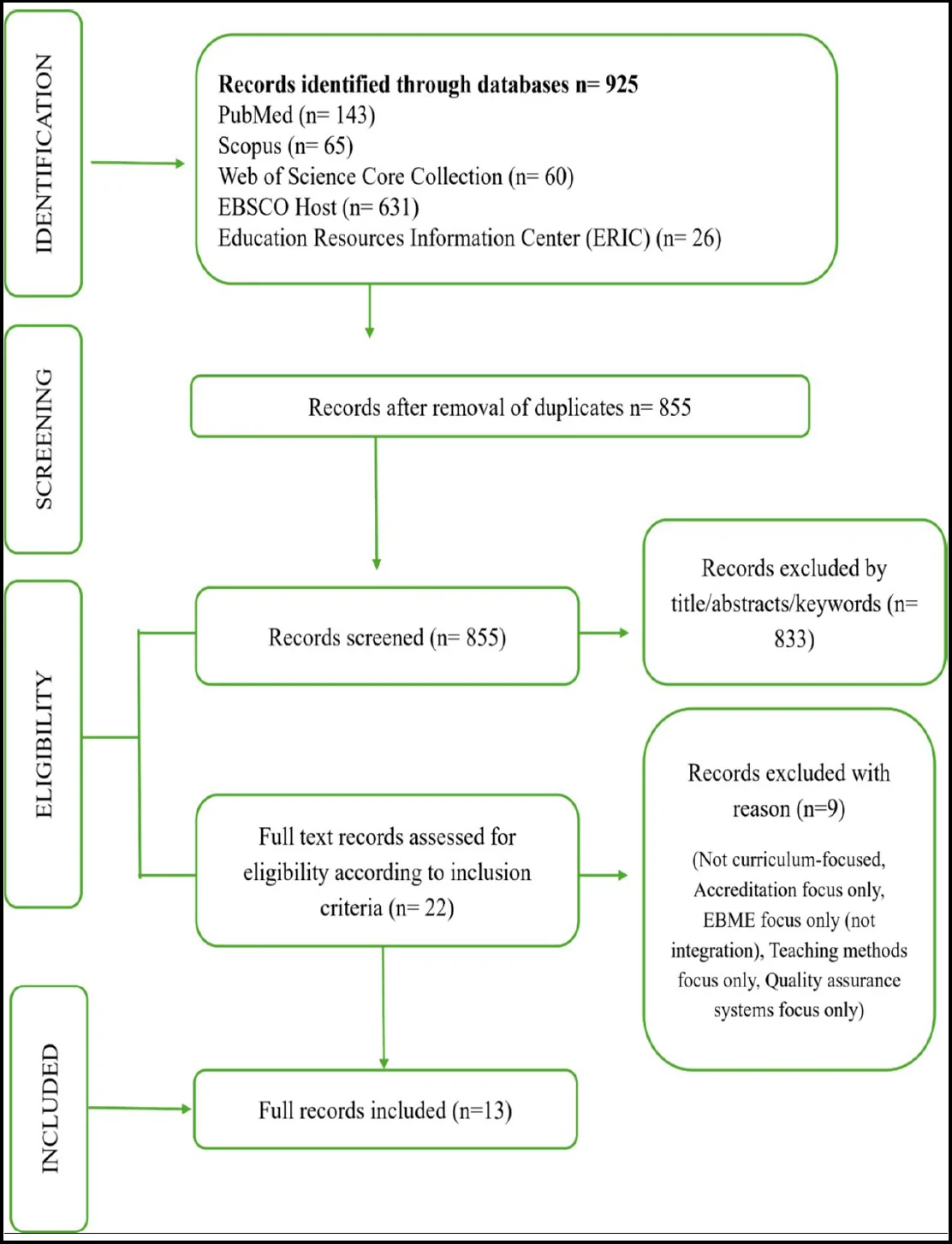
PRISMA-ScR Flowchart.

- Articles published between 1st January 2010 and 31 December 2025.
- Original research articles published in peerreviewed journals.
- Fulltext articles available in the English language.
- Studies conducted in LMICs settings.
- Studies that examined the implementation of undergraduate medical curricula with explicit integrative features, including horizontal, vertical, systems-based, spiral, or similarly structured integrated designs.
- Studies reporting challenges related to the implementation of UGME curricula.

### Studies were excluded if they:

- Fell outside the 1 January 2010 – 31 December 2025 publication window.
- Were not original peer-reviewed research, such as editorials, commentaries, or conference abstracts without full text.
- Had no full text available or were not published in English.
- Were conducted outside LMIC settings.
- Did not involve an explicit curricular integration feature e.g., studies centered solely on accreditation, evidence-based medical education without an integration component, teaching methods, or quality assurance systems.
- Did not report challenges related to UGME curriculum implementation.

The starting year of 2010 was selected for this review as it coincides with a global shift in medical education from conventional, disciplinebased curricula towards integrated curricular models.^15^ These integrated models are interrelated dimensions of the same integration continuum with competency-based, modular, and spiral designs unfolding complementary frameworks through which integration is organized, delivered and sequenced.^16^ While curriculum integration had been discussed earlier, the Lancet Commission on Health Professionals for a New Century in 2010 consolidated these ideas and reported the importance of integrated curricula, healthsystem relevance, and outcomebased educational models.^17^ These priorities were subsequently reinforced through international accreditation standards, most notably by the World Federation for Medical Education (WFME)^18^ which positioned integrated curriculum as core quality standards.

All search results retrieved from the databases were exported to the Mendeley reference manager for compilation.

### Stage four: Data charting:

The lead author developed a structured Excel-based charting form, designed around the review questions to keep consistent data extraction. It recorded basic publication details, i.e., country, World Bank income classification, study design, and data source alongside curriculum-specific information, the model of integration (horizontal, vertical, modular, or competency-based), which program phases it affected, and how it was implemented. The form, furthermore, tracked the stakeholders involved, the barriers and facilitators each study reported, and any implementation outcomes or process indicators. It also described system-level factors, including policy and accreditation context, assessment alignment, infrastructure and resources, monitoring and evaluation, and equity. The co-authors cross-checked this data for completeness and consistency.

### Stage five: Compiling and summarizing the findings:

The data was organized around the three research questions using two complementary approaches. Descriptively, curriculum models, implementation settings, stakeholder involvement, and system-level features were compared across studies. Thematically, the focus shifted to recurrent barriers, facilitators, and contextual influences that apply to number of cases. The predefined coding fields that covered domains such as governance, faculty development, assessment, resources, stakeholder engagement, quality assurance, and equity supported systematic data extraction. The subsequent inductive approach yielded six broad implementation themes, each comprising recurring sub-themes (Table-II) through iterative comparison and consolidation. This method assisted in comparison of implementation challenges and strategies across diverse settings while preserving contextual nuance. Findings are reported in accordance with PRISMAScR guidance (Fig.1).

## RESULTS

A total of 925 studies were identified, of which 855 were screened and 833 excluded at the title/abstract stage; twenty-two articles underwent full-text review, with nine excluded for not directly addressing UGME curriculum implementation, yielding thirteen included studies (Fig.1).

### Characteristics of UGME curriculum implementation studies in LMICs:

Table-I summarizes the characteristics of the thirteen studies, including setting, design, integration model, UGME phase focus, and participant groups across Sri Lanka, Pakistan, Syria, Brazil, Uganda, Vietnam, Egypt, Iran, Cambodia, Ethiopia, India and Bhutan.

**Table-I:** Characteristics of included studies on UGME curriculum implementation in LMICs.

Author, Year	Country	Institution type	Study design	Curriculum model type	UGME phase focus	Participants
Ranasinghe et al. 2011	Sri Lanka	Public university	Qualitative	Integrated modular curriculum	Whole UGME	Graduates, faculty
Ali & Baig, 2012	Pakistan	Public medical colleges	Mixed methods	Integrated community-oriented Problem-based curriculum	Years 1–5 (UGME)	Faculty, students
Kayyal & Gibbs, 2012	Syria	Public medical faculty	Qualitative	Integrated curriculum	Whole UGME	Academic leaders, faculty
Alves et al. 2013	Brazil	Public universities	Qualitative	Horizontal & vertical integrated curriculum	Whole UGME	Academic leaders, faculty
Kiguli et al. 2014	Uganda	Consortium of schools	Qualitative	Integrated competency-based curriculum	Whole UGME	Academic leaders, faculty
Tran et al. 2022	Vietnam	Public university	Qualitative	Integrated competency-based curriculum	Whole UGME	Academic leaders, faculty
Badrawi et al. 2023	Egypt	National reform	Mixed Methods	Integrated competency-based curriculum	UGME + internship	Students, faculty
Khanipoor et al. 2024	Iran	Public university	Qualitative	Horizontal & vertical integrated curriculum	Whole UGME	Students, faculty
Lim et al. 2024	Cambodia	National system	Qualitative	Integrated competency-based curriculum	UGME	Academic leaders, Regulators
Bakhshi et al. 2024	Pakistan	National multi-institution	Mixed methods	Integrated curriculum	Years 1–5 (UGME)	Academic leaders
Gebremickael et al. 2024	Ethiopia	Public universities	Mixed methods	Integrated competency-based curriculum	Pre-clerkship/ clerkship UGME	Faculty, coordinators
Dhandapani et al. 2025	India	Government medical college	Quantitative	Integrated competency-based curriculum	Whole UGME (MBBS)	Faculty
Tenzin et al. 2025	Bhutan	New medical school	Qualitative	Integrated competency-based curriculum	Whole UGME (MBBS)	Governance, ministries

***Notes on Table I terminology:*** i. Whole UGME: Entire undergraduate medical program (typically Years 1–5/6, depending on country), ii. Graduates: Medical graduates who completed the curriculum, reflecting retrospectively on their training experience., iii. Academic leaders: Institutional leadership roles such as deans, department heads, or curriculum committee members. iv. UGME + Internship: The full training continuum, including the internship year; however, directly surveyed participants were students and teaching faculty, with internship-related findings drawn from supporting institutional reports. v. Regulators: National governing bodies overseeing medical education policy (e.g., Ministry of Health), vi. Pre-clerkship/clerkship UGME: Distinguishes the preclinical, basic-science years (pre-clerkship) from the clinical, hospital-based training years (clerkship) within the undergraduate program.

### RQ1: Types of implementation challenges in UGME in LMICs:

Six thematic categories of implementation challenges were identified. Table-II summarizes the themes, contributing studies, frequency of reporting, and sub-themes.

**Table-II:** Types of curriculum implementation challenges in UGME across LMICs.

Challenging theme	Studies reporting the theme	No. of studies (n/N)	Sub-themes
Resources & infrastructure constraints	Ali 2012 (Pakistan); Kayyal & Gibbs 2012 (Syria); Alves 2013 (Brazil); Kiguli 2014 (Uganda); Tran 2022 (Vietnam); Badrawi 2023 (Egypt); Khanipoor 2024 (Iran); Lim 2024 (Cambodia); Bakhshi 2024 (Pakistan); Gebremickael 2024 (Ethiopia); Dhandapani 2025 (India); Tenzin 2025 (Bhutan)	12/13	Faculty shortage, deficient skills labs, limited educational technologies, insufficient clinical training space, financial constraints, resources underutilization
Governance, organizational and policy barriers	Ali 2012 (Pakistan); Kayyal & Gibbs 2012 (Syria); Alves 2013 (Brazil); Kiguli 2014 (Uganda); Tran 2022 (Vietnam); Badrawi 2023 (Egypt); Lim 2024 (Cambodia); Bakhshi 2024 (Pakistan); Gebremickael 2024 (Ethiopia); Khanipoor 2024 (Iran); Dhandapani 2025 (India); Tenzin 2025 (Bhutan)	12/13	Policy-practice gaps, organizational culture barriers, variable leadership engagement, limitations in community-based educational settings
Faculty capacity and development needs	Ali 2012 (Pakistan); Kayyal & Gibbs 2012 (Syria); Kiguli 2014 (Uganda); Tran 2022 (Vietnam); Badrawi 2023 (Egypt); Khanipoor 2024 (Iran); Lim 2024 (Cambodia); Bakhshi 2024 (Pakistan); Gebremickael 2024 (Ethiopia); Dhandapani 2025 (India); Tenzin 2025 (Bhutan)	11/13	Limited faculty preparedness, faculty development gaps, increased workload, resistance to change
Assessment challenges	Ranasinghe 2011 (Sri Lanka); Ali 2012 (Pakistan); Kayyal & Gibbs 2012 (Syria); Alves 2013 (Brazil); Kiguli 2014 (Uganda); Tran 2022 (Vietnam); Badrawi 2023 (Egypt); Khanipoor 2024 (Iran); Lim 2024 (Cambodia); Gebremickael 2024 (Ethiopia); Bakhshi 2024 (Pakistan)	11/13	Assessment-outcome misalignment, assessment-examination framework gaps, need for standardized assessment structures
Curriculum integration issues	Ranasinghe 2011 (Sri Lanka); Ali 2012 (Pakistan); Alves 2013 (Brazil); Badrawi 2023 (Egypt); Bakhshi 2024 (Pakistan)	5/13	Content overload, horizontal/vertical integration challenges
Instructional and training settings constraints	Ali 2012 (Pakistan); Lim 2024 (Cambodia); Gebremickael 2024 (Ethiopia)	3/13	Predominant conventional teaching, gaps in patient-centered learning, variable learning access

***NOTE:*** Sub-themes represent recurring patterns identified within each broad theme during synthesis.

### RQ2: Similarities and contextspecific differences in implementation challenges across LMICs:

The review also examined how these challenges manifested across LMICs to identify common and context-specific patterns. Resource and infrastructure constraints, governance and coordination barriers, and misalignment between curricular intentions and assessment were reported consistently across studies.^1^,^19^-^30^ The contextspecific challenges in implementing centralized national reforms were reported in Egypt,^1^ Uganda,^22^ Cambodia,^26^ Ethiopia.^28^ The challenges related to institution/organizationallevel culture, coordination amongst systems and leadership engagement were highlighted in Sri Lanka.^19^, Syria,^21^ Vietnam,^24^ and Iran,^25^ Pakistan^20^ and Ethiopia^28^ reported rural and communitybased training supervision shortages, logistical constraints, and variable learning access. Uganda^23^ and Ethiopia^28^ described absolute shortages of faculty, infrastructure, and clinical training spaces while Brazil^22^ and Iran^25^ reported challenges in optimizing and coordinating existing resources.

### RQ3: Gaps in the current literature regarding the implementation of integrated UGME curricula in LMICs:

Four evidence gaps were identified during data charting and synthesis: implementation outcomes, rural/community-based teaching contexts, stakeholder perspectives, and assessment processes. Table-III summarizes these domains and the extent to which they were reported in the included studies.

**Table-III:** Evidence gaps in the literature on implementation of integrated UGME curricula in LMICs (n = 13).

Gap area	Adequate reporting	Studies addressing the area (n)	Studies not addressing or superficially addressing the area (n)	Nature of the evidence gap
Implementation outcomes	Reporting of implementation fidelity, learner performance trends, or documented curricular impact beyond structural change	3	10	Most studies focused on curriculum design or reform processes, with limited evaluation of whether integrated curricula were implemented as intended or led to measurable educational outcomes
Rural / community implementation context	Detailed description of implementation in rural or peripheral sites (e.g., supervision, transport, scheduling, site readiness)	2	11	Communitybased or rural learning was frequently stated as an objective but rarely accompanied by operational detail on implementation logistics or sustainability
Stakeholder breadth (learners and frontline supervisors)	Inclusion of students and frontline clinical supervisors/preceptors alongside faculty or leadership perspectives	3	10	The literature predominantly reflects leadership or faculty viewpoints, with limited attention to learner experiences or supervisory roles in daytoday implementation
Assessment implementation processes	Operational detail on how assessment innovations (e.g., OSCEs, WBA, programmatic assessment) were implemented, managed, and used	5	8	While assessment reform was commonly mentioned, most studies lacked processlevel detail on feasibility, workflow, data use, or alignment with integrated curricula

***Abbreviations:*** UGME (Undergraduate Medical Education), OSCE (Objectively Structured Clinical Examination), WBA (WorkplaceBased Assessment).

## DISCUSSION

This review synthesized thirteen studies on the implementation of integrated UGME curricula in LMICs. The findings collectively suggest that implementation barriers are rarely attributable to curriculum design itself but tend to be a recurring mismatch between curricular ambition and the institutional capacity, governance, and assessment systems needed to sustain it. The six identified themes were not independent problems. They were distinct aspects of the same underlying challenge.

### RQ1: Types of implementation challenges in UGME in LMICs:

The findings reveal that barriers to implementing integrated UGME curricula in LMICs arise from system-level implementation capacity rather than deficiencies in curricular design. Constraints in resources and infrastructure capacity, particularly teaching faculty numbers, skills-lab readiness, educational technologies, and clinical training spaces appear to restrict most of the achievable curriculum reforms.^1^,^19^-^29^ Notably, the capacity constraints are not exclusive feature of LMICs. In the United Kingdom, Geary et al., and Thornton et al,^31^,^32^ reported that limitations in clinical placements can hinder reform goals under different resource contexts.

Ineffective policy translation, disjointed coordination among departments and training locations, and varying levels of leadership involvement can result in inconsistent implementation, even when the curriculum aims are well-defined.^1^,^19^-^29^ Similar findings from three Dutch medical schools reported by Velthuis et al.^33^ show that ambiguous governance and uneven leadership can impede the achievement of desired goals. Another issue is the need for faculty capacity and development, which is similar to the demands of integrated and competency-based approaches on teaching roles, feedback practices, and cross-disciplinary collaboration.^1^,^19^,^20^,^22^-^29^ This is in line with extensive reviews by Steinert et al.,^34^ who emphasized that while sustainable curricular reform requires longterm organizational strategies and a supportive institutional culture, faculty development plays a critical role in strengthening educators’ teaching and changemanagement capabilities. Likewise, in Pakistan, Bakhshi et al.^27^ found that leadership support for institutional reforms was limited due to persistent faculty development issues and resource constraints underscoring the need for sustained dialogue between faculty and academic administrative sections such as Medical Education departments to align reform intent with on ground capacity.

Misaligned assessment emerged as another major barrier. When high-stakes examinations do not reflect integrated outcomes, teaching may revert to disciplinary silos and exam-focused learning.^1^,^19^-^27^,^30^ From a theoretical perspective, WijnenMeijer et al.^2^ related that vertical integration is a coordinationintensive educational philosophy, underscoring the need for alignment among faculty readiness, resources, governance, and assessments.

### RQ2: Similarities and contextspecific differences in implementation challenges across LMICs:

The underlying need for alignment among curricular outcomes, faculty readiness, resources, and governance was consistent among all thirteen studies^1^,^19^-^30^, however, the challenges varied with each context’s governance structure and resource base. Resource-constrained settings faced shortages in faculty, infrastructure, and clinical placement more often,^22^,^27^ while relatively better-resourced settings struggled with coordination and optimization of existing systems.^21^,^24^ As noted by Velthuis et al.^33^, such contexts may get benefit from processintegration strategies aimed at improving curricular coherence and crossdisciplinary integration. In addition, settings undergoing topdown national reforms tend to struggle with policy translation and crossinstitutional coordination,^1^,^22^,^25^,^27^ whereas institutionlevel reforms often face challenges related to organizational culture and local leadership.^20^,^23^,^24^,^30^ Tran et al., similarly, reported these issues and recommended joint planning mechanisms, governance restructuring, performancebased evaluation and comprehensive qualitymonitoring processes to strengthen implementation.^24^

Rural and community-based training settings faced additional challenges related to supervision, logistics, and uneven access to learning opportunities.^19^,^25^,^27^ The WHO guideline on rural health workforce development proposed structured preparation and support for rural supervisors, scaffolding students’ transition into rural placements and community-engaged rural training pathways for trainees.^35^ Overall, these distinctions suggest that adaptation should be guided less by country income alone and more by the practical configuration of governance and training ecology.

### RQ3: Gaps in the current literature regarding the implementation of integrated UGME curricula in LMICs:

This review unveiled that the included literature more often describes structural and organizational conditions than the enactment of integration in practice. Across the studies, reporting rarely extends to implementation outcomes such as curriculum fidelity, learner performance patterns, or broader curriculum impact, leaving the relationship between reform activities and educational effects limited.^1^,^19^,^20^,^22^-^27^,^30^ This gap might be explained through a study by Alias A et al., which found that medical schools struggle to generate outcomelevel data because of faculty workload, time constraints, documentation burden and immature institutional infrastructure for evaluation.^12^ Another gap is related to implementation in rural and communitybased training contexts, where description on distributed clinical learning in remote settings is limited.^19^,^25^,^27^

This reflects systemlevel constraints in rural sites such as inadequate infrastructure, limited supervision capacity, heavy clinical workload, and uneven coordination. The WHOsupported rural frameworks advocate the strategies such as coinvestment models between universities and rural health services, structured planning tools for site readiness, dedicated leadership roles for rural planning, and continuous monitoring and evaluation of rural learning quality to strengthen community-based learning quality.^35^

Finally, the literature provided limited coverage of the full range of stakeholders involved in curriculum delivery, particularly undergraduate students and frontline clinical supervisors/preceptors.^1^,^19^-^30^ This pattern may reflect the emphasis of studies on faculty and academic leaders, while students and supervisors can be more difficult to access due to service commitments and limited availability for research participation.^36^ It also offered few operational accounts of how assessment innovations were implemented (e.g., OSCE logistics, workplace-based assessment workflows, and the use of assessment information in progression decisions). This indicates that documenting assessment operations and decisionmaking processes may be less feasible in contexts where assessment infrastructure, data systems, protected time, and administrative support are inadequate.^12^ On the whole, literature included in this review documented limited operational realities and outcomes of integration. Addressing these gaps would strengthen understanding of how integrated curricula are enacted across sites and how educational impact could be assessed.

### Strengths:

The strengths of this review include a systematic, five-database search aligned with PRISMA-ScR guidance, independent two-phase screening by a four-member team having strong inter-rater reliability (Cohen’s k = 0.84), and synthesis across thirteen diverse LMIC settings offering a broader cross-regional perspective than single-country studies typically may provide.

### Limitations:

Limitations include restriction to Englishlanguage, peerreviewed studies that may result in a potential loss of relevant evidence from nonEnglishspeaking LMIC context, limiting the cultural and regional diversity of perspectives. Since scoping reviews seek to provide an overview of the extent and range of available evidence rather than pinpoint every contextual and procedural detail, summarizing diverse studies into broad categories can reduce the contextual detail presented in individual papers. Lastly, it was challenging to directly compare implementation processes across contexts because the included research varied in design, scope, and reporting depth. Notwithstanding, this review provides an organized overview of the existing body of data and emphasizes context-sensitive, capacity-oriented, and governance-aligned implementation strategies.

## CONCLUSION

This scoping review entails that implementing integrated UGME curricula in LMICs is less a matter of curriculum design than of institutional and systemic capacity. The shared challenges identified across the thirteen studies included resource and infrastructure constraints, weak governance, inadequate faculty development, and assessment misalignment. Yet, how these challenges unfolded depended heavily on each country’s reform structure, institutional culture, and rural training context. In addition, important evidence gaps remain around implementation outcomes, assessment strategies, stakeholder perspectives, and rural settings.

### Recommendations:

Future research should prioritize measuring implementation fidelity and outcomes, capture student and supervisor perspectives, and examine rural training contexts specifically. On the other hand, future reform efforts should prioritize context-responsive governance, faculty support, and assessment alignment.
